# TP3, an antimicrobial peptide, inhibits infiltration and motility of glioblastoma cells via modulating the tumor microenvironment

**DOI:** 10.1002/cam4.3005

**Published:** 2020-04-07

**Authors:** Ying‐Fa Chen, Po‐Chang Shih, Hsiao‐Mei Kuo, San‐Nan Yang, Yen‐You Lin, Wu‐Fu Chen, Shiow‐Jyu Tzou, Hsin‐Tzu Liu, Nan‐Fu Chen

**Affiliations:** ^1^ Department of Neurology Kaohsiung Chang Gung Memorial Hospital and Chang Gung University College of Medicine Kaohsiung Taiwan; ^2^ Center for Parkinson's Disease Kaohsiung Chang Gung Memorial Hospital and Chang Gung University College of Medicine Kaohsiung Taiwan; ^3^ UCL School of Pharmacy University College London London UK; ^4^ Department of Marine Biotechnology and Resources National Sun Yat‐sen University Kaohsiung Taiwan; ^5^ Center for Neuroscience National Sun Yat‐sen University Kaohsiung Taiwan; ^6^ Department of Internal Medicine E‐DA Hospital and College of Medicine I‐SHOU University Kaohsiung Taiwan; ^7^ Department of Orthopedic Surgery Ping‐Tung Christian Hospital Pingtung Taiwan; ^8^ Department of Neurosurgery Kaohsiung Chang Gung Memorial Hospital and Chang Gung University College of Medicine Kaohsiung Taiwan; ^9^ Department of Neurosurgery Xiamen Chang Gung Hospital Xiamen Fujian China; ^10^ Department of Nursing Kaohsiung Armed Forces General Hospital Kaohsiung Taiwan; ^11^ Institute of Medical Science and Technology National Sun Yat‐Sen University Kaohsiung Taiwan; ^12^ Department of Medical Research Hualien Tzu Chi Hospital Buddhist Tzu Chi Medical Foundation Hualien Taiwan; ^13^ Division of Neurosurgery Department of Surgery Kaohsiung Armed Forces General Hospital Kaohsiung Taiwan; ^14^ Department of Neurological Surgery Tri‐Service General Hospital National Defense Medical Center Taipei Taiwan

**Keywords:** cell mobility, glioblastoma multiforme, infiltration, TP3, tumor microenvironment

## Abstract

Glioblastoma multiforme (GBM) is a cancer of the central nervous system with limited therapeutic outcomes. Infiltrating cancer cells are the contributing factor to high GBM malignancy. The intracranial brain cancer cell infiltration is a complex cascade involving adhesion, migration, and invasion. An arsenal of natural products has been under exploration to overcome GBM malignancy. This study applied the antimicrobial peptide tilapia piscidin 3 (TP3) to GBM8401, U87MG, and T98G cells. The cellular assays and microscopic observations showed that TP3 significantly attenuated cell adhesion, migration, and invasion. A live‐cell video clip showed the inhibition of filopodia protrusions and cell attachment. Probing at the molecular levels showed that the proteolytic activities (from secretion), the mRNA and protein expression levels of matrix metalloproteinases‐2 and ‐9 were attenuated. This result strongly evidenced that both invasion and metastasis were inhibited, although metastatic GBM is rare. Furthermore, the protein expression levels of cell‐mobilization regulators focal adhesion kinase and paxillin were decreased. Similar effects were observed in small GTPase (RAS), phosphorylated protein kinase B (AKT) and MAP kinases such as extracellular signal‐regulated kinases (ERK), JNK, and p38. Overall, TP3 showed promising activities to prevent cell infiltration and metastasis through modulating the tumor microenvironment balance, suggesting that TP3 merits further development for use in GBM treatments.

## Graphical abstract

We first describe the anti‐cancer activities of the antimicrobial peptide tilapia piscidin 3 which inhibits infiltration and motility of glioblastoma multiforme cells.

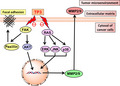

## 1 INTRODUCTION

Glioblastoma multiforme (GBM), a WHO‐classified grade IV histological malignancy, is a central nervous system (CNS) tumor that is generally considered to arise from glial cells.^1^ Glioblastoma multiforme constitutes the majority of aggressive CNS tumors.^2^ Approximately 90% of clinically diagnosed GBM cases are primary tumors, whereas the remainder are secondary glioblastomas developed from lower‐grade astrocytic tumors.^3^ Although cancer research has made great improvements on the treatments of most types of cancer, generally, patients with GBM have a median survival of approximately 15 months.^4^, ^5^ The current commonly used GBM treatment modality of maximal surgical resection, followed by radiotherapy and a small‐molecule alkylating drug temozolomide, is suffering limited therapeutic outcomes with recurrence often occurred.^5^ Hence, new therapy against GBM is definitely needed.

The cancer malignancy of GBM is subjected to the ability of tumors to infiltrate into adjacent and/or distant tissues and the balance of tumor microenvironment (TME) along the vascular tracks. The invasion, commonly considered to result from cancer cell infiltration, enables cancer cells to escape from the tumor site of origin, leading to metastasis. The occurrence of infiltration is related to the degradation of the extracellular matrix (ECM) within the TME. In particular, the process breaks down the basement membrane of the ECM, resulting in the disturbance of the TME.^6^ The ECM is a three‐dimensional (3D) extracellular network consisting of macromolecules (eg proteoglycan, proteins including collagens, etc) that support cell mass^7^ and surround blood vessels in all tissues.^8^ In cancer, this 3D network can restrict the growth of tumors to some extent and define borders for solid tumors. However, this is not the case with brain cancer. Glioblastoma multiforme cells are known to center in the white matter of cerebral hemispheres; however, they are well‐documented to show malignant infiltration into neighboring brain tissues and/or the opposite hemisphere.^9^, ^10^ This infiltration makes no clear borders of the tumor mass, largely ablating the chance for a complete surgical resection and radiation therapy. This results in poor prognosis and the possibility of tumor recurrence increases accordingly.^11^ In brain, the basement membrane is also an important component for the blood‐brain barrier (BBB). The breakdown of the basement membrane pathologically is linked to the disruption of the BBB,^12^ which increases the chance of GBM migration as GBM cells often migrate along vasculature structures.^8^

The malignant infiltration of GBM has been reported to result from the aberrant induction of signaling pathways that lead to the disruption of the TME balance. In this respect, signaling axes centered on mitogen‐activated protein kinases (MAPKs), ATK or focal adhesion kinase (FAK) have been strongly implicated in causing the aggressive transformation.^13^, ^14^, ^15^, ^16^, ^17^, ^18^, ^19^ These axes are well‐documented to regulate cell adhesion and mobility^17^, ^18^, ^20^ and/or the expression of matrix metalloproteinases (MMP)2 and MMP9.^21^, ^22^, ^23^, ^24^, ^25^, ^26^, ^27^, ^28^ Both MMPs are proteolytic enzymes responsible for the degradation of collagens and fibronectin, the major components of the basement membrane of the ECM.^29^

An arsenal of natural products has been explored to develop drugs for use in GBM treatments. Tilapia pisicidin (TP3), isolated from Nile tilapia (*Oreochromis niloticus*), was identified as a marine antimicrobial peptide (AMP) that has characteristics of being amphipathic, poly‐cationic, and α‐helical at physiologic pH.^30^ This peptide, consisting of 23 amino acid residues, has shown inhibition on both Gram‐positive and ‐negative bacteria^30^, ^31^, ^32^ through disrupting bacterial membrane. In addition, it has displayed inhibition on a hand‐foot‐and‐mouth disease virus.^33^ To our best knowledge, TP3 had not been applied in the field of anti‐cancer. Interestingly, it has been reported that AMPs have considerable potentials in the anti‐tumor application.^34^, ^35^ In this study, we applied TP3 to U87MG and GBM8401 cells, to understand its potential in the prevention of the GBM malignant transformation at cellular and molecular levels.

## 2 MATERIALS AND METHODS

### 2.1 Preparation of TP3

Tilapia pisicidin (FIHHIIGGLFSVGKHIHSLIHGH) was provided by Professor Jyh‐Yih Chen's laboratory (Academia Sinica of Taiwan). The stock solution of TP3 was prepared using a solution of phosphate buffered saline (PBS) at pH 7.2. Prior to the experiments, the TP3 stock solution was protected from light and stored at −20°C.

### 2.2 Cell culture

The glioblastoma cell lines GBM8401, U87MG, and T98G cells were provided by Prof. Zhi‐Hong Wen (Department of Marine Biotechnology and Resources, National Sun Yat‐sen University, Kaohsiung, Taiwan), and utilized as described previously.^36^ Briefly, the U87MG and T98G cells were maintained using Minimum Essential Medium (Thermo Fisher Scientific), while the GBM8401 cells were cultured in Roswell Park Memorial Institute medium 1640 medium (Thermo Fisher Scientific). The above media contained 50 U/mL penicillin, 50 mg/mL streptomycin (Thermo Fisher Scientific), and 10% heat‐inactivated fetal bovine serum (FBS) (Thermo Fisher Scientific). A humidified atmosphere of 5% CO_2_ and 95% room air at 37°C was applied to the culturing of the cells. Subculture was carried out every 2‐3 days. The resulting cells were used directly for the subsequent experiments. All cellular operations were performed in a sterile environment.

### 2.3 Cell adhesion evaluation

The suspension of the glioblastoma cells (3 × 10^4^ cell/ well) was seeded in collagen‐coated 24‐well plates which were supplemented with specified concentrations of TP3. Each condition was conducted in triplicate and incubated for 8 hours. Subsequently, the unattached cells were gently removed with media, and the attached cells were stained with 3‐(4,5‐dimethylthiazol‐2‐yl)‐2,5‐diphenyltetrazolium bromide (MTT) for optical density reading. The absorbance was recorded at 570 nm and determined using an ELISA plate reader (epoch; Bio Tek Instruments, Inc).

### 2.4 Live‐cell tomographic imaging

The live‐cell imaging was conducted using the procedures as described in our previous study.^37^ Briefly, GBM8401 and U87MG cells were seeded onto the glass bottom of a 3.5 cm dish overnight, followed by 24 hours incubation with TP3 at concentrations of 0 (as a control) and 10 μmol/L. The visualization of the 3D morphology and adhesion and localization of quantum dots were performed by interferometric detection using a tomographic, holographic 3D microscope Nanolive (3D Cell Explorer). The time‐lapse video was captured every 15 minutes for a shooting time of 5 hours, and each image was recorded. Subsequently, the STEVE software (3D Cell Explorer) was used for image processing.

### 2.5 Transwell chamber migration assay

The migration abilities of GBM8401, U87MG, and T98G cells following TP3 treatments were evaluated using the transwell chamber migration assay. Each condition was repeated three times. Transwell inserts with 8 µmol/L pore size were selected (Corning Inc). Glioblastoma cells were seeded on top of the filter membrane, at a density of 2 × 10^4^ using 1% FBS containing specified TP3 concentrations. The lower chamber was added 10% FBS as a chemo‐attractant to induce cell migration. Following 16‐hour incubation, with care, cotton‐tipped applicators were used to remove residual solution and remaining cells from the upper part of the membrane. The migrated cells on the other side of membrane were washed with 1× PBS, fixed using 4% paraformaldehyde and 10% Giemsa‐stained for 25 minutes. A phase‐contrast microscope (Leica Microsystems) was utilized for observations of the lower part of membrane, and the images were captured using a SPOT CCD RT‐slider integrating camera (Diagnostic Instruments). In the transwell migration capture image, we used the ImageJ analysis software to evaluate the number of migrated cells from the images of three randomly selected regions in every transwell insert.

### 2.6 Transwell chamber invasion assay

The procedures and consumables for the transwell chamber invasion assay were followed for the transwell chamber migration assay, except for the filter membrane necessary pre‐coating. At the bottom of the membrane was pre‐coated with matrigel (Corning Inc) as a mimic of the ECM prior to the invasion assay.

### 2.7 Gelatin zymography

The secretion of MMPs by glioblastoma cells was assayed by 0.1% gelatin‐SDS‐PAGE zymography. Briefly, the conditioned media were collected from TP3‐treated glioblastoma cells for 24 hours and assayed for cell counter by the trypan blue method (Bio‐Rad). Aliquots of conditioned media were subjected to separation with 10% sodium dodecyl sulfate‐polyacrylamide gel electrophoresis (SDS‐PAGE) containing 0.1% type A gelatin (Sigma). After electrophoresis, gel was washed twice with 2.5% Triton X‐100, incubated in a buffer containing 40 mmol/L Tris‐HCl, pH 8.0, 10 mmol/L CaCl_2_ at 37°C for 12‐24 hours, stained with 0.25% Coomassie Blue R‐250 in 50% methanol and 10% acetic acid for 1 hour, and de‐stained with 10% acetic acid and 20% methanol. The gelatinolytic regions by the MMPs were visualized as white bands in blue background. Visualization of white bands was completed using camera imaging. LabWorks 4.0 software (UVP LLC) was used to conduct relative densitometry analysis of bands.

### 2.8 Immunoblotting analysis

Following 24 hours of TP3 treatments, the glioblastoma cells were lysed. Protein concentrations from cell lysates were determined using the Bradford method (Bio‐Rad), followed by the conduct of SDS‐PAGE (8%‐12%) for separation. The resulting SDS‐PAGE gels were transferred onto the polyvinylidene difluoride (Millipore) membrane which was then blocked with 5% non‐fat milk. Primary antibodies specific to MMP2 (1:1000; Merck), MMP9 (1:500; Abcam), p‐AKT (1:1000; Cell Signaling), AKT (protein kinase B, 1:1000; Cell Signaling), FAK (1:500; Invitrogen), paxillin (1:1000; Cell Signaling), RAS (small GTPase, 1:1000; Cell Signaling), p‐ERK (1:1000; Cell Signaling), ERK (extracellular signal‐regulated kinases, 1:1000; Cell Signaling), p‐JNK (1:1000; Cell Signaling), JNK (1:1000; Cell Signaling), p‐p38 (1:1000; Cell Signaling), p38 (1:1000; Cell Signaling), and β‐actin (internal control) (1:2000, cat: A5441; Sigma‐Aldrich) were applied onto the membrane which was incubated at 4°C overnight. On the following day, the membrane was incubated with secondary antibodies conjugated with horseradish peroxidases at 37°C for 1 hour. The generated signals were recorded using enhanced chemiluminescence (ECL‐kit; Millipore). Visualization of bands was completed using UVP BioChemi imaging (UVP LLC). LabWorks 4.0 software (UVP LLC) was used to conduct relative densitometry analysis of bands. Subsequently, the membrane samples were re‐stripped, re‐blocked, and re‐used for monoclonal antibodies against β‐actin as the internal control for protein loading.

### 2.9 Real‐time polymerase chain reaction

The glioblastoma cells were treated with TP3 at various concentrations for 24 hours, and washed with ice‐cold PBS. Then, total RNA extraction from the cells was performed using the Qiagen RNeasy Mini kit (Qiagen) according to the manufacturer's instructions. A spectrophotometer was utilized to determine concentrations and yields of the samples, and to confirm their quality based on ratios of absorbance at 260 nm over 280 nm. The reverse transcription of RNA samples into complementary DNA (cDNA) was completed using the iScriptTM cDNA Synthesis Kit (Bio‐Rad). To obtain the single strand of cDNA, a 20 µL mixture of 1 µg of total RNA, 4 µL of 5× iScript reaction mix (containing oligo(dT) and random hexamer primers, reaction buffer with dNTP), iScript reverse transcriptase, RNAse inhibitors, and nuclease‐free water was prepared. The mixture was reacted at 25°C for 5 minutes and 46°C for 20 minutes, followed by running at 95°C for 1 minute to render the enzyme inactivated for termination. Subsequently, the synthesized cDNA was subjected to a quantitative reverse transcription‐polymerase chain reaction (qRT‐PCR) analysis using a CFX‐96 real‐time PCR system (Bio‐Rad) for amplification and detection. The PCR procedure is as follows: one cycle of 95°C for 10 minutes, 45 cycles of 95°C for 15 seconds, 62°C for 5 seconds, and 72°C for 20 seconds. The primers for human target and housekeeper genes (see Table 1) were designed using Primer 3 software, from Integrated DNA Technologies. The relative gene mRNA expression of the target gene was calculated using the following formula: mRNA relative expression = 2^−ΔΔCt^ = 2^−(ΔCt(target)−ΔCt(housekeeper))^, where Ct is the threshold cycle, and ΔCt is the difference between Ct_(target)_ and Ct_(housekeeper)_. β‐actin is representative housekeeper.

**TABLE 1:** Human gene primers used in quantitative real‐time PCR

Name	Gene no.		Gene length (bps)	Primer sequence 5′‐3′	Amplicon (bps)	Annealing temperature
MMP2	NM_004530.6	1982		F: CAACTACGATGATGACCGCAA	140	62
				R: GTGTAAATGGGTGCCATCAGG		
MMP9	NM_004994.3	2124		F: TTGACAGCGACAAGAAGTGG	179	62
				R: GCCATTCACGTCGTCCTTAT		
β‐actin	NM_001101.5	1127		F: TCACCCACA CTGTGCCTATCTACGA	295	62
				R: CAGCGGAACCGCTCATTGCCAATGG		

Abbreviations: MMP2, matrix metallopeptidase 2; MMP9, matrix metallopeptidase 9; PCR, polymerase chain reaction.

### 2.10 Statistical analysis

The results were expressed as means ± SE. Data were analyzed using the two‐tailed Student's *t* test analysis. *P* < .05 were considered significant.

## 3 RESULTS

### 3.1 TP3 significantly ablates glioblastoma cell adhesion and affects filopodia protrusions but slightly decreases cell proliferation

**FIGURE 1:**
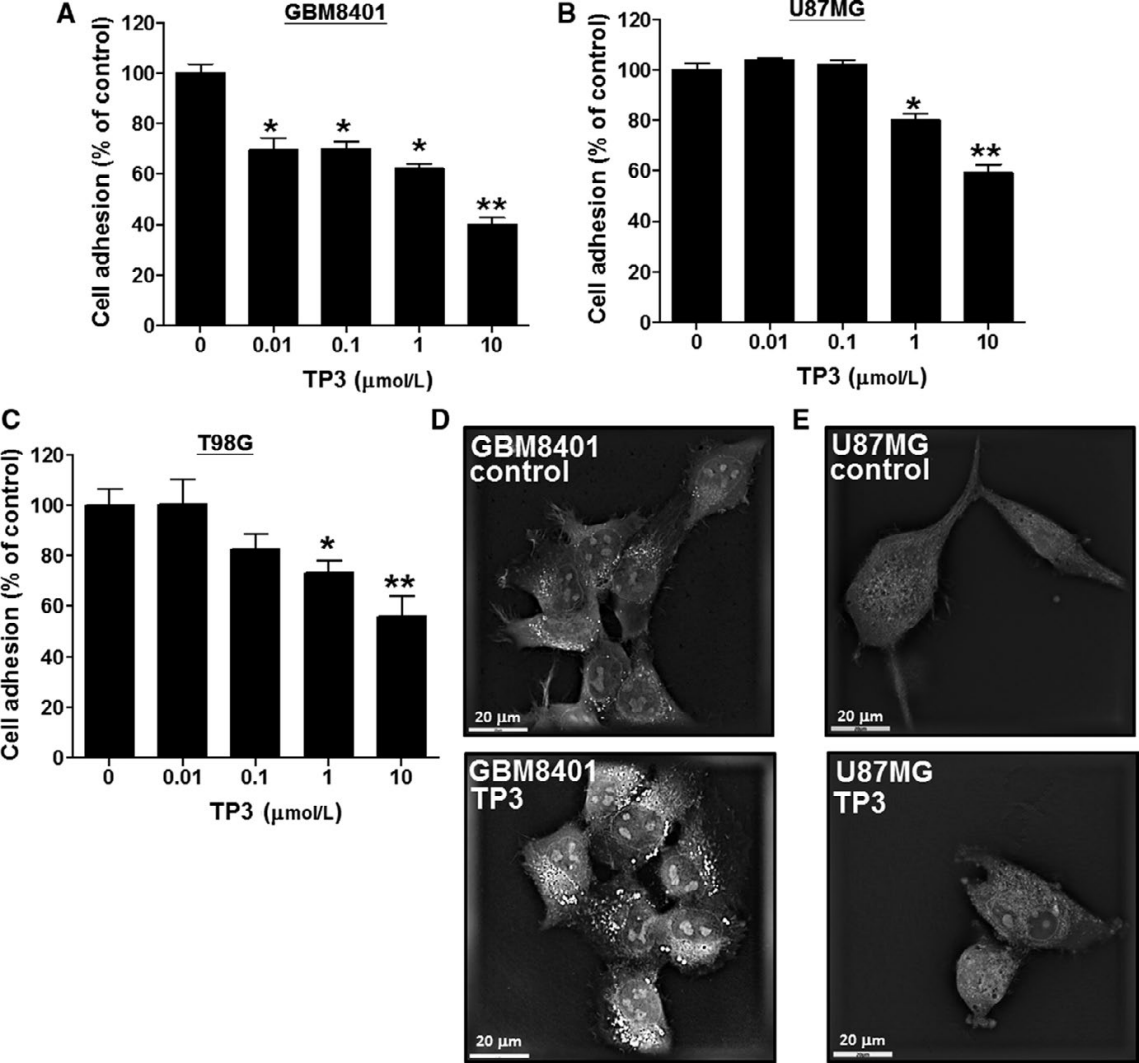
Effects of tilapia piscidin 3 (TP3) on the cell adhesion and filopodia protrusions in GBM8401 and U87MG cells. The suspension of the glioblastoma cells was seeded in collagen‐coated plates overnight. A, GBM8401, (B) U87MG, and (C) T98G cells were treated with TP3 at various concentrations in pre‐coating collagen plates for 8 h, followed by measuring the cell adhesion using the MTT assay. Each bar represents mean ± SE (n = 6) from three independent experiments; *P < .05; **P < .01, relative to the control. D, GBM8401 and (E) U87MG cells were treated with 10 µmol/L concentrations of TP3 for 24 h and photographed by interferometric detection using a tomographic, holographic microscope

The adhesion onto the ECM is believed to be a step essential for the migration of infiltrating cells and for the establishment of the secondary tumor mass after invasion.^38^, ^39^ The promotion of anti‐adhesion is therefore a plausible way to treat cancer. Various concentrations of TP3 were applied to collagen‐coated plates pre‐cultured with glioblastoma cells, followed by 8‐hour incubation. The loss of attachment to collagen that occurred with some cells was assumed to result from the loss of adhesion. In GBM8401 cells, the adhesion responses were significantly reduced to 69.3 ± 4.1%, 70.2 ± 2.4%, 65.4 ± 2.8%, and 40.0 ± 2.8% of the control level at TP3 concentrations of 0.01, 0.1, 1, and 10 µmol/L, respectively (Figure 1A). In U87MG cells, the cell adhesion levels were significantly reduced to 80.1 ± 2.4%, and 59.3 ± 3.2% of the control level at TP3 concentrations of 1 and 10 µmol/L, respectively (Figure 1B). In T98G cells, the cell adhesion levels were significantly reduced to 73.3 ± 4.8% and 56.0 ± 8.2% of the control level at TP3 concentrations of 1 and 10 µmol/L, respectively (Figure 1C). To visualize the morphological changes, particularly filopodia protrusions, a live‐cell imaging study was conducted using 0 and 10 µmol/L of TP3. Filopodia are thin, spike‐like projections at the leading edge of cells constructed by cytoskeleton filaments. These outstretching filopodia structures are assumed to probe the environment and to guide the direction of cell adhesion and migration.^40^, ^41^ In this imaging study, GBM8401 and U87MG cells were photographed by interference imaging using a tomographic, holographic microscope at magnification (600×) after treated with 10 μmol/L TP3 for 24 hours (Figure 1D,E). Additionally, GBM8401 cells were subjected to performing live cells time‐lapse imaging experiments, capturing cell images once every 15 minutes for 5 hours, and recording the dynamic changes of the edge extension (Video S1). We found that the extension at the leading edge of the cell membrane was prominent before the addition of TP3. Following the addition of TP3 (10 μmol/L), the leading edge was indented, resulting from the outermost cell surface collapsed which left the cell membrane to be ebb tide‐like. We also determined cell viability under 24 hours of TP3 treatments in GBM8401, U87MG, and T98G cells lines using the MTT stain method. At TP3 concentrations of 1 and 10 μmol/L, GBM8401 cell viability were significantly reduced to 86.8 ± 2.0% and 78.3 ± 1.6% of the control (100 ± 5.3%) level, respectively (Figure S1A); U87MG cell viability was significantly reduced to 73.1 ± 4.2% (10 μmol/L) of the control (100 ± 3.0%) level (Figure S1B), while T98G cell viability was significantly reduced to 89.0 ± 4.2% (1 μmol/L) and 83.7 ± 4.6% (10 μmol/L) of the control (100 ± 1.6%) level (Figure S1C). Taken together, these results suggest that TP3 can inhibit the cell adhesion of the glioblastoma cells at low doses (0.01 μmol/L for GBM8401, 1 μmol/L for U87MG, and 1 μmol/L for T98G) with slight inhibition on their cell viability (1 μmol/L for GBM8401, 10 μmol/L for U87MG, and 1 μmol/L for T98G). Without TP3 treatments, the filopodia bodies appeared markedly at the cell edges in both cell lines, whereas they were contracted following TP3 treatments.

### 3.2 Cell migration of glioblastoma cells is significantly inhibited using TP3 treatments

**FIGURE 2:**
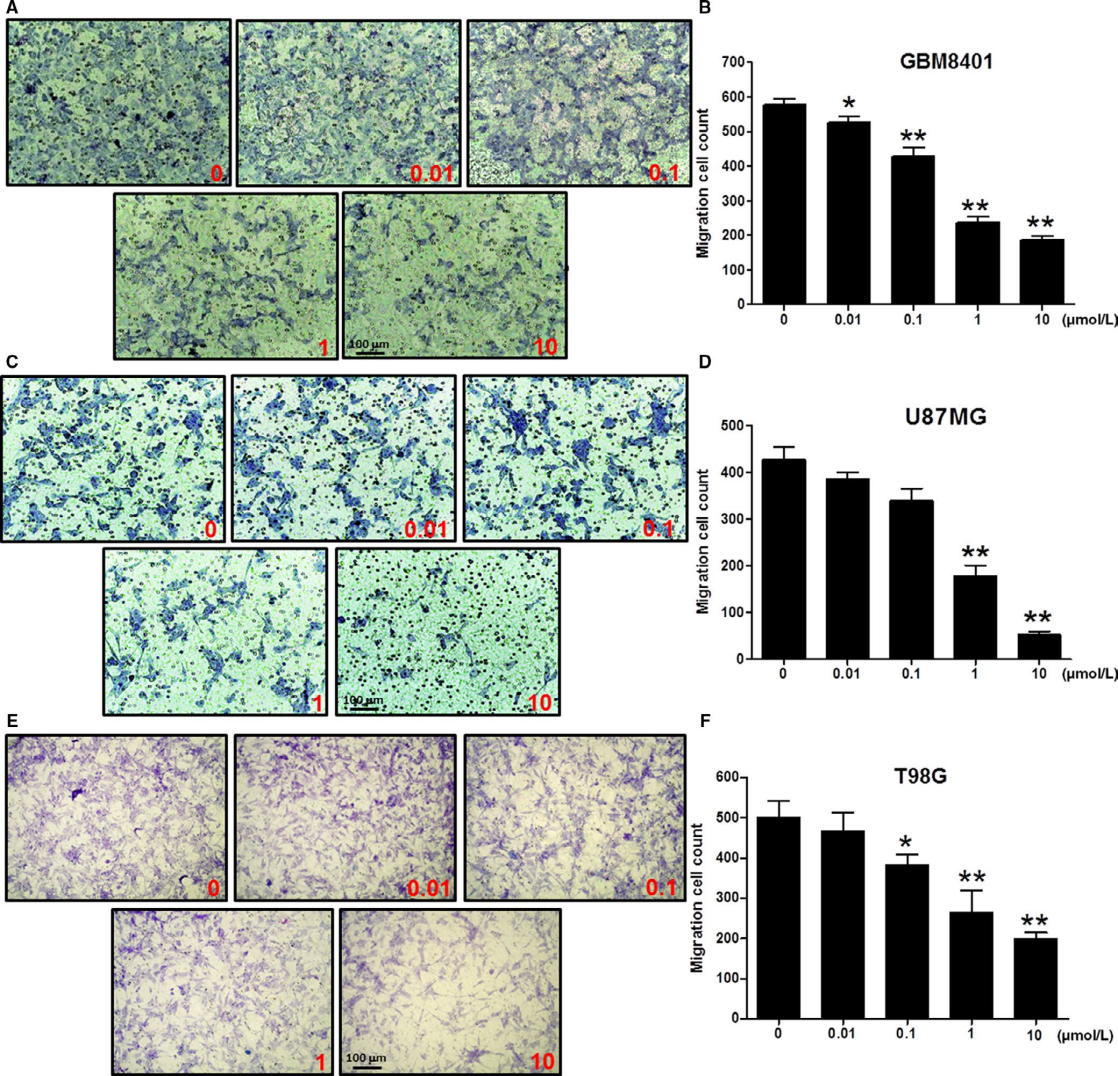
Effects of tilapia piscidin 3 (TP3) on cell migration of the glioblastoma cells at various concentrations. A, The profile of GBM8401 cells treated with TP3 at 0.01, 0.1, 1, and 10 µmol/L for 24 h prior to evaluations for the chemotactic potential. The photographs present the cell migration morphologies using bright field microscopy. B, Quantification of the migration cell count of GBM8401 cells. C, The profile of U87MG cells treated with TP3 at concentrations of 0.01, 0.1, 1, and 10 µmol/L for 24 h prior to evaluations for the chemotactic effect. The photographs present the cell migration morphologies using bright field microscopy. D, Quantification of the migration cell count of U87MG cells. E, The profile of T98G cells treated with TP3 at concentrations of 0.01, 0.1, 1, and 10 µmol/L for 24 h prior to evaluations for the chemotactic effect. The photographs present the cell migration morphologies using bright field microscopy. F, Quantification of the migration cell count of T98G cells. Each bar represents mean ± SE from three independent experiments; *P < .05; **P < .01, relative to the control

The transwell assay was used to analyze the effects of TP3 on the anti‐migration in glioblastoma cells. The experimental results showed that GBM8401 (Figure 2A), U87MG (Figure 2C), and T98G (Figure 2E) cells treated with TP3 at various concentrations inhibited their migration profiles. The migrated cells were counted and analyzed using the ImageJ soft counters. At TP3 concentrations of 0.01, 0.1, 1, and 10 μmol/L, the numbers of GBM8401 migrated cells were significantly reduced to 527.3 ± 9.7, 427.3 ± 15.5, 236.7 ± 10.5, and 186.3 ± 6.6 cells of the control (576.3 ± 10.3 cells) level, respectively (Figure 2B). In U87MG cells, the numbers for were significantly reduced to 178.0 ± 22.5 (1 μmol/L) and 52.3 ± 6.7 cells (10 μmol/L) of the control (426.3 ± 27.0 cells) level (Figure 2D), while the numbers for T98G cells were significantly reduced to 381.3 ± 15.8 (0.1 μmol/L), 262.7 ± 32.5 (1 μmol/L), and 198.0 ± 9.7 cells (10 μmol/L) of the control (501.0 ± 23.3 cells) level (Figure 2F). The results demonstrated that the migration of both GBM8401, U87MG, and T98G cells was significantly attenuated after 24 hours of TP3 treatments to a point as low as 0.01 µmol/L (*P* < .05), 1 µmol/L (*P* < .01) and 0.1 µmol/L (*P* < .05), respectively.

### 3.3 TP3 treatments significantly attenuate the invasion potential of glioblastoma cells

**FIGURE 3:**
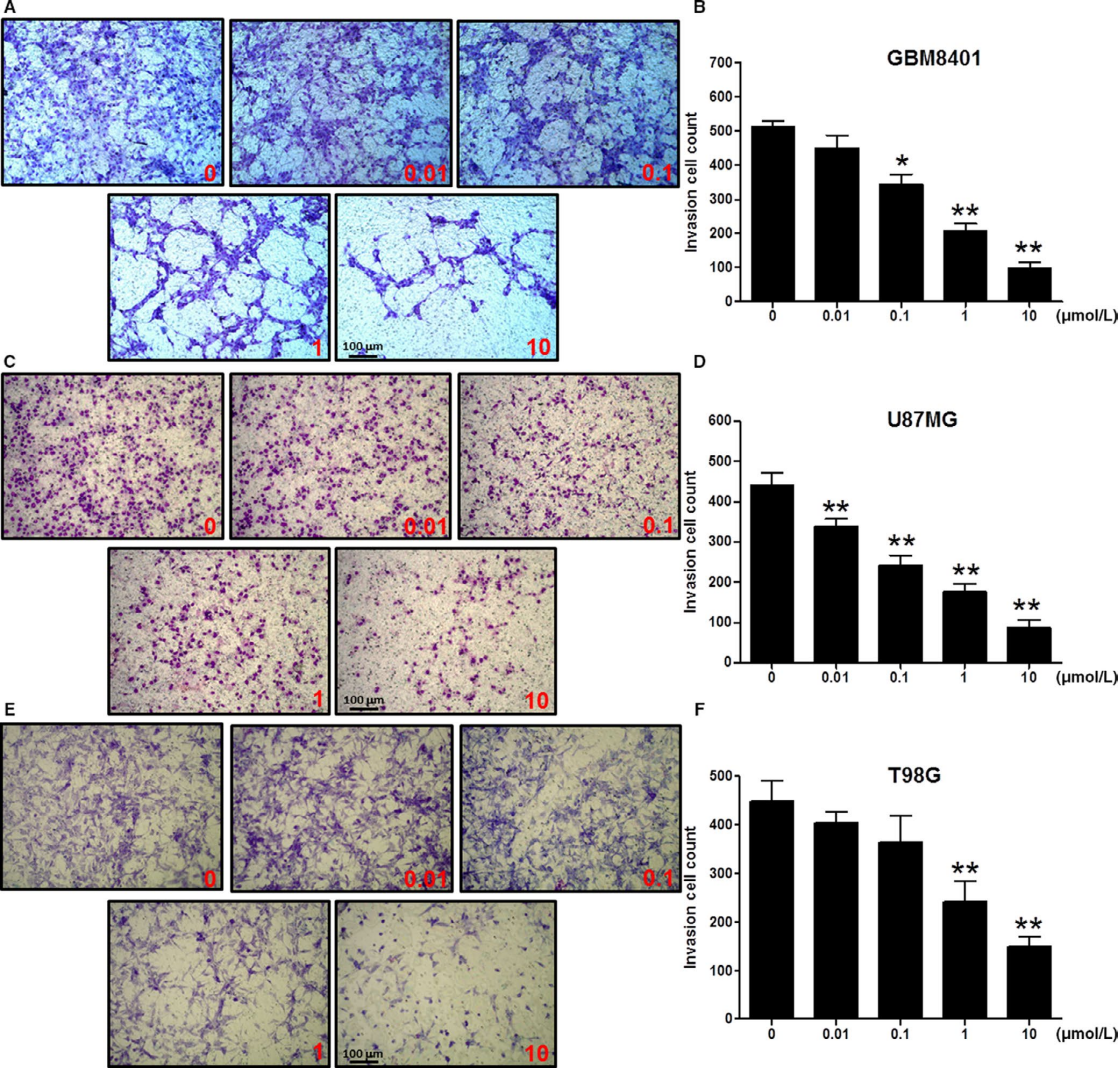
Effects of tilapia piscidin 3 (TP3) on the invasion of the glioblastoma cells at varying concentrations. A, The profile of GBM8401 cells treated with TP3 of 0.01, 0.1, 1, and 10 µmol/L for 24 h prior to evaluations for the invasion potential. The photographs present the cell invasion morphologies using bright field microscopy. B, Quantification of the invasion cell count of GBM8401 cells. C, The profile of U87MG cells treated with TP3 of 0.01, 0.1, 1, and 10 µmol/L for 24 h prior to evaluations for the invasion effect. The photographs present the cell invasion morphologies using bright field microscopy. D, Quantification of the invasion cell count of U87MG cells. E, The profile of T98G cells treated with TP3 of 0.01, 0.1, 1, and 10 µmol/L for 24 h prior to evaluations for the invasion effect. The photographs present the cell invasion morphologies using bright field microscopy. F, Quantification of the invasion cell count of T98G cells. Each bar represents mean ± SE from three independent experiments; *P < .05; **P < .01, relative to the control

Cell invasion is a more advanced step in the progression of cancer development. The anti‐invasion ability of TP3 was demonstrated using the transwell invasion assay in which Matrigel was utilized to mimic the basement membrane of the ECM. The experimental results of GBM8401 (Figure 3A), U87MG (Figure 3C), and T98G (Figure 3E) cells treated with various concentrations of TP3 showed their inhibited invasion profiles. The invaded cells were counted and analyzed using the ImageJ soft counters. At TP3 concentrations of 0.1, 1, and 10 µmol/L, the numbers of GBM8401 invaded cells were significantly reduced to 344.3 ± 17.0, 206.7 ± 12.9, and 97.3 ± 11.0 cells of the control (511.7 ± 9.9 cells) level, respectively (Figure 3B). In U87MG cells, the numbers were significantly reduced to 338.7 ± 11.0 (0.01 µmol/L), 241.7 ± 14.2 (0.1 µmol/L), 175.7 ± 11.8 (1 µmol/L), and 87.0 ± 11.0 cells (10 µmol/L) of the control (441.3 ± 17.1 cells) level (Figure 3D), while for T98G cells the numbers were significantly reduced to 242.0 ± 24.3 (1 µmol/L) and 149.0 ± 11.6 cells (10 µmol/L) of the control (448.3 ± 24.3 cells) level (Figure 3F). Overall, our findings showed that the invasion of GBM8401 and U87MG cells were inhibited significantly at sub‐micromolar concentrations after 24 hours of TP3 treatments.

### 3.4 The ECM disassemblers MMP2 and MMP9 are significantly down‐regulated after TP3 treatments

**FIGURE 4:**
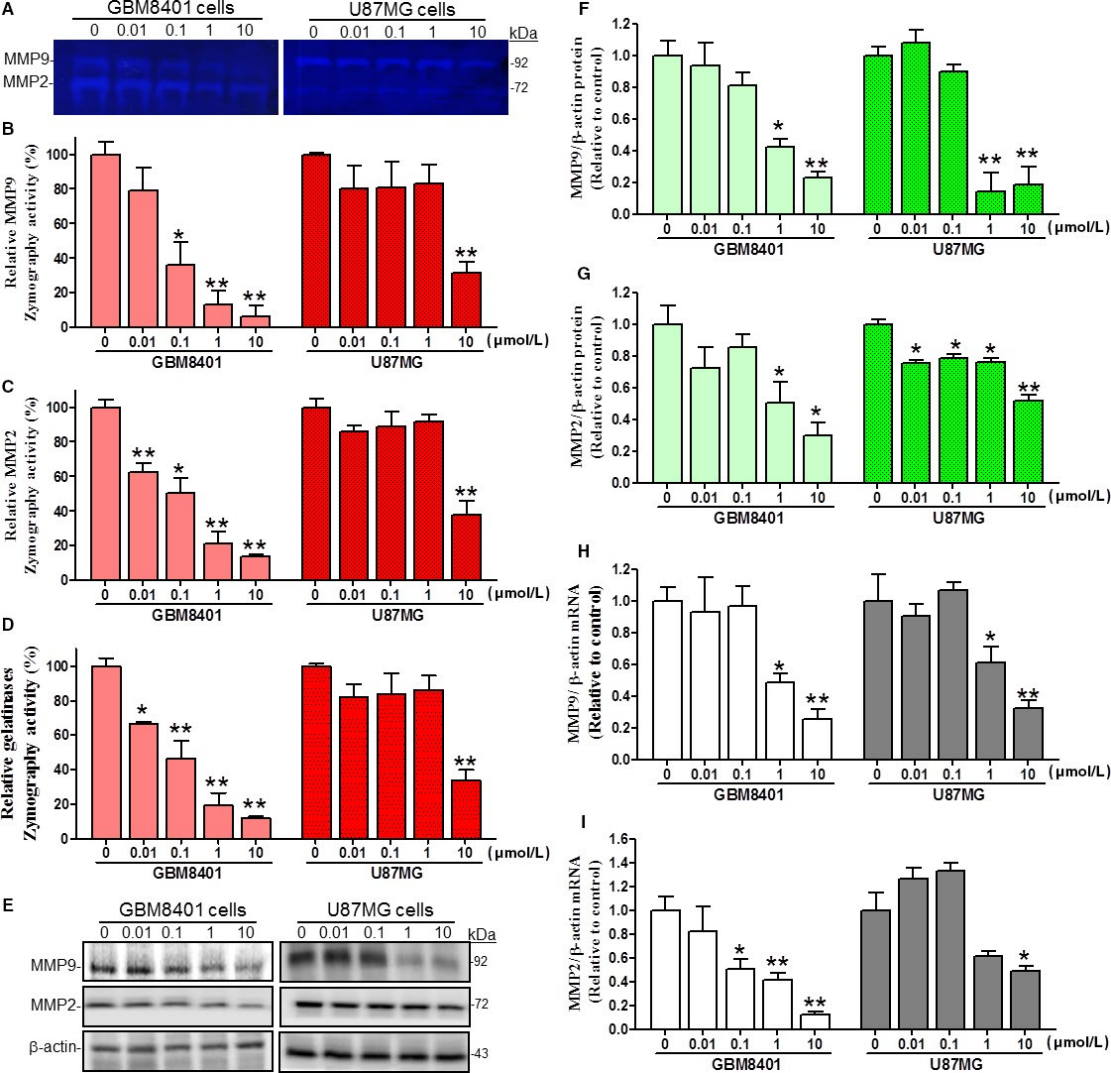
Effects of TP3 treatments on the proteolytic activities, secretion and expression levels of MMP2 and MMP9 in GBM8401 and U87MG cells after 24 h. A, The effect of TP3 on MMP‐2 and MMP‐9 gelatinase activities in cultured media of GBM8401 and U87MG cells. After treated with TP3 at different concentrations for 24 h, the cultured media of GBM8401 and U87MG cells were normalized with respective cell numbers for analyses of MMP‐2 and MMP‐9 activity patterns via gelatin–SDS‐PAGE zymography. B, Quantification of band intensities of relative MMP9 zymography activities in both cell lines using ImageJ software. C, Quantification of band intensities of relative MMP2 zymography activities in both cell lines using ImageJ software. D, Quantification of band intensities of relative total gelatinases (MMP9 and MMP2) zymography activities in both cell lines using ImageJ software. E, The effect of TP3 on endogenous MMP‐2 and MMP‐9 protein levels in GBM8401 and U87MG cells. After treated with TP3 for 24 h, the MMP‐2 and MMP‐9 protein levels in cell extracts from GBM8401 and U87MG cells were determined using western blot analyses. The β‐actin levels were analyzed as a protein loading control. F, Quantification of band intensities of relative MMP9 protein levels in GBM8401 and U87MG cells using ImageJ software. G, Quantification of band intensities of relative MMP2 protein levels in GBM8401 and U87MG cells using ImageJ Software. H, The effects of TP3 on MMP9 mRNA levels in GBM8401 and U87MG cells. After treated with TP3 for 24 h, MMP9 mRNA levels in GBM8401 and U87MG cells were determined using qRT‐PCR analyses. The MMP9 mRNA levels were expressed as ratios of melting temperature for MMP9 compared to those of β‐actin. I, The effects of TP3 on MMP2 mRNA levels in GBM8401 and U87MG cells. After treated with TP3 for 24 h, MMP2 mRNA levels in GBM8401 and U87MG cells were determined using qRT‐PCR analyses. The MMP2 mRNA levels were expressed as ratios of melting temperature for MMP2 relative to those of β‐actin. Each bar represents mean ± SE from three independent experiments; *P < .05; **P < .01, relative to the control. MMP2, matrix metallopeptidase 2; MMP9, matrix metallopeptidase 9; qRT‐PCR, quantitative real‐time PCR; TP3, tilapia piscidin 3

Cancer spreading from the site of origin to nearby and/or distant areas of tissues is a process that requires the initial degradation of the basement membrane of the ECM. Matrix metalloproteinases, a family of zinc‐containing endopeptidases, mediate selective degradation of the ECM. To determine the effect of TP3 on MMPs secretion, conditioned media from GBM8401 and U87MG cells treated with TP3 peptide were normalized with respective cell numbers, then subjected to gelatin‐zymography analysis. The gelatin‐zymography profile was used to show the effects of TP3 on the protein expression levels using markers associated with MMPs, including MMP2 (72 kDa) and MMP9 (92 kDa) in GBM8401 and U87MG cells (Figure 4A). Our results showed that MMP9 zymography activities were significantly decreased to 36.1 ± 13.2%, 12.9 ± 8.5%, and 6.6 ± 5.9% of the control (100.0 ± 6.9%) level at 0.1, 1, and 10 µmol/L, respectively, in GBM8401 cells. However, for U87MG cells, the significant decrease in MMP9 zymography activity occurred at a higher concentration of 10 µmol/L, to 31.5 ± 6.6% of the control (100.0 ± 0.7%) level (Figure 4B). Similarly, MMP2 zymography activities were significantly decreased to 62.6 ± 5.1%, 50.2 ± 8.8%, 21.3 ± 6.9%, and 13.9 ± 1.2% of the control (100.0 ± 4.0%) level at 0.01, 0.1, 1, and 10 µmol/L, respectively in GBM8401 cells. Nonetheless, in U87MG cells, the significant decrease in MMP2 zymography activity occurred at a higher concentration of 10 µmol/L, to 38.0 ± 8.1% of the control (100.0 ± 5.1%) level (Figure 4C). In addition, we analyzed combinational gelatinases activities (MMP2 and MMP9 zymography) using the results of Figure 4A in both cell lines. The combinational gelatinases zymography activities in GBM8401 cells were significantly decreased to 66.7 ± 1.1%, 46.6 ± 9.9%, 19.2 ± 7.3%, and 12.1 ± 0.8% of the control (100.0 ± 4.7%) level at 0.01, 0.1, 1, and 10 µmol/L, respectively. Nevertheless, in U87MG cells, the significant decrease in the combined gelatinases zymography activity occurred at the concentration of 10 µmol/L, to 33.8 ± 6.0% of the control (100.0 ± 1.5%) level (Figure 4D). These observations were further supported by western blot analyses, which show the effects of TP3 on the protein expression levels using markers associated with MMPs, including MMP2 (72 kDa) and MMP9 (92 kDa). The bands related to β‐actin (43 kDa) were utilized as an internal control (Figure 4E). At TP3 concentrations of 1 and 10 µmol/L, MMP9 protein was significantly declined to 0.4 ± 0.1 and 0.2 ± 0.0 of the control (1.0 ± 0.1) level, respectively, in GBM8401 cells. For U87MG cells, MMP9 protein was significantly declined to 0.2 ± 0.1 (1 and 10 µmol/L) of the control (1.0 ± 0.1) level (Figure 4F). At TP3 concentrations of 1 and 10 µmol/L, MMP2 protein was significantly declined to 0.5 ± 0.1 and 0.3 ± 0.1 of the control (1.0 ± 0.1) level, respectively, in GBM8401 cells. For U87MG cells, MMP2 protein was significantly declined to 0.8 ± 0.0 (0.01, 0.1, and 1 µmol/L) and 0.5 ± 0.1 (10 µmol/L) of the control (1.0 ± 0.0) level (Figure 4G). Similarly, quantitative reverse transcription polymerase chain reaction (qRT‐PCR) analyses indicated that treatments with TP3 at various concentrations affected the mRNA expression of MMP2 and MMP9 in GBM8401 and U87MG cells. For example, TP3 treatments decreased the MMP9 mRNA expression in GBM8401 and U87MG cells by approximately 60%‐70% of the control at 10 µmol/L (Figure 4H). Tilapia piscidin 3 treatments decreased the MMP2 mRNA levels in GBM8401 and U87 cells by approximately 50%‐80% of the control at 10 µmol/L (Figure 4I). Collectively, these data suggested that TP3 perturbed the release of MMP2/9 and their protein and mRNA levels in GBM8401 and U87MG cells.

### 3.5 The MAPKs, AKT, and FAK/paxillin are attenuated following TP3 treatments in glioblastoma cells

**FIGURE 5:**
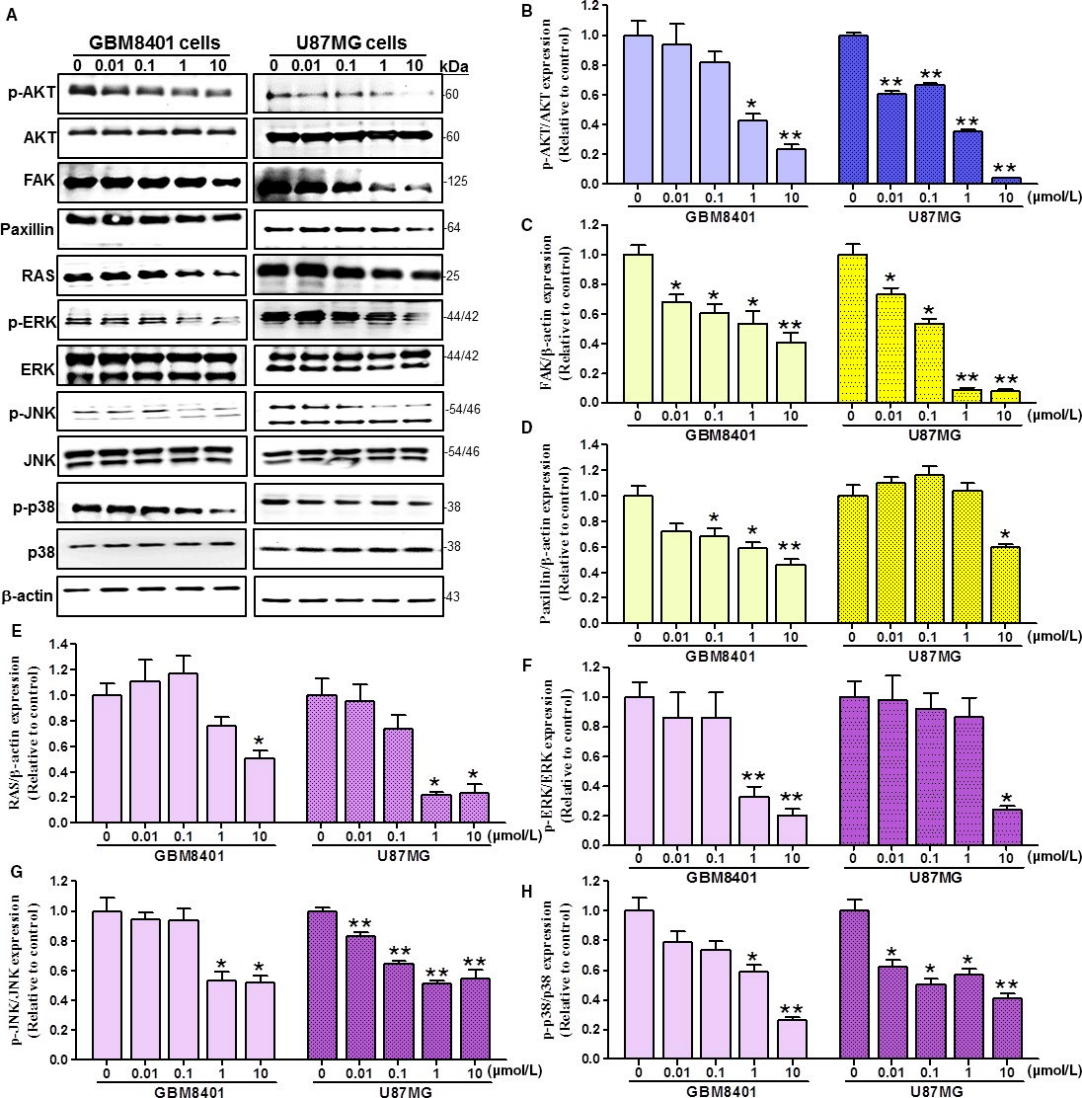
Effects of TP3 treatments on the expression levels of AKT, FAK, paxillin, RAS, ERK, JNK, p38 and phosphorylated forms of the kinases in GBM8401 and U87MG cells after 24 h. A, GBM8401 and U87MG brain cancer cells were treated with the indicated dose of TP3 for 24 h. However, cells lysates and proteins were loaded to western blot analysis using antibodies of AKT, FAK, paxillin, RAS, p‐ERK, p‐JNK, p‐p38, ERK, JNK, p38, and β‐actin. The β‐actin levels were analyzed as the protein loading control. The AKT (B), FAK (C), paxillin (D), RAS (E), p‐ERK/ERK (F), p‐JNK/JNK (G), and p‐p38/p38 (H) protein levels were quantified by ImageJ software, normalized with respective β‐actin levels, and presented as a normalization of the control. Each bar represents mean ± SE. *P < .05; **P < .01, relative to the control. ERK, JNK, p38, mitogen‐activated protein kinases; FAK, focal adhesion kinase; GBM, glioblastoma multiforme; MMP2, matrix metallopeptidase 2; MMP9, matrix metallopeptidase 9; p‐ERK, phosphorylated ERK; p‐JNK, phosphorylated JNK; p‐p38, phosphorylated p38; TP3, tilapia piscidin 3

Mitogen‐activated protein kinases have been reported to be constitutively phosphorylated (p‐MAPKs), which correlates with poor prognosis in patients.^42^, ^43^, ^44^, ^45^ Similarly, aberrantly phosphorylated AKT (p‐AKT) protein levels have been found in most GBM tumor samples and cell lines.^46^ We have therefore immunoblotted the MAPKs (ERK, JNK, and p38 kinases) and AKT in addition to a MAPKs upstream regulator RAS. Moreover, mobility and migration‐associated proteins FAK and paxillin were likewise immunoblotted, demonstrated as the western blot protein band profiles with the β‐actin as an internal control (Figure 5A). The FAK is a binding partner with paxillin responsible for focal adhesion (FAs) at the cell front, while the paxillin proteins are localized at FAs, which are contact surfaces between the ECM and cytoskeleton.^18^, ^47^ Our results demonstrated that the ratios of p‐AKT vs AKT were decreased upon the administration of TP3 in a dose‐dependent manner, with a significant decrease of approximately 75% in GBM8401 cells and a significant decrease of approximately 95% in U87MG cells at 10 µmol/L (Figure 5B). Moreover, western blot analyses revealed that with exposure to 10 µmol/L TP3 for 24 hours, the FAK levels were declined by about a twofold decrement over the control in GBM8401 cells, but by about a 10‐fold decrement in U87MG cells (Figure 5C). The paxillin expression quantities were ablated significantly at 10 µmol/L, with a decrease of approximately 50% in GBM8401 cells and a decrease of approximately 40% in U87MG cells (Figure 5D). In both cell lines, the RAS expression quantities were reduced in each administered concentration, with a decrease of approximately 50% in GBM8401 cells and a decrease of approximately 75% in U87MG cells at 10 µmol/L (Figure 5E). We also observed the TP3‐induced ablation of the ratios of p‐MAPKs versus MAPKs, inclusive of p‐ERK/ERK, p‐JNK/JNK, and p‐p38/p38. The expression quantities of p‐ERK/ERK, p‐JNK/JNK and p‐p38/p38 were attenuated in each administered concentration in both cell lines. At 10 µmol/L of TP3 in GBM8401 and U87MG cells, p‐ERK/ERK ratios decreased by ~80% and ~75% (Figure 5F), respectively; p‐JNK/JNK ratios were ablated ~50% in both GBM8401 and U87MG cells (Figure 5G); p‐p38/p38 ratios were ablated ~75% and 60%, respectively (Figure 5H). Taken together, these results showed that TP3 decreased mobility and migration in the glioblastoma cells via the MAPKs, AKT, and FAK/paxillin signaling pathways.

## 4 DISCUSSION

Glioblastoma multiforme is the most aggressive cancer that originates in the brain and accounts for 15% of brain tumors.^48^, ^49^ The pathogenicity in most cases remains unclear with less than 3%‐5% of the survival rate after 5 years. Without treatments, most patients can only survive for about 3 months.^50^, ^51^ Malaka et al reported that in newly diagnosed glioblastoma patients, the use of anti‐angiogenic therapies did not significantly improve overall survival,^52^ indicating that new treatments and drugs are necessary to overcome this issue.

Tilapia piscidin 3 is a marine‐derived antimicrobial peptide, with a molecular weight of about 2.5 kDa. They are resistant to bacteria and difficult to induce bacterial resistance. In addition, TP3 is important to the development of antibacterial drug research. To our best knowledge, there was no anti‐cancer research on TP3, while TP4 has been reported to have anti‐cancer activities.^6^, ^53^ The sequence between TP3 (FIHHIIGGLFSVGKHIHSLIHGH) and TP4 (FIHHIIGGLFSAGKAIHRLIRRRRR) is similar and both peptides have amphiphilic, α‐helical, cationic characteristics, suggesting that TP3 may have the potential to treat glioblastoma. Herein, activities of TP3 against GBM in GBM8401, U87MG, and T98G cells were first described. The microscopic observations and cell adhesion assay of cellular responses to TP3 demonstrated that this peptide was capable of ablating cell adhesion, which was unlikely to result from TP3‐induced cell death. Following the evaluation of anti‐invasion, significant inhibitions on migration and invasion were found at sub‐micromolar concentrations in both cell lines. Moreover, the downregulated levels of MMP2 and MMP9 after TP3 treatments have shown its anti‐invasion and anti‐mobility potentials.

**FIGURE 6:**
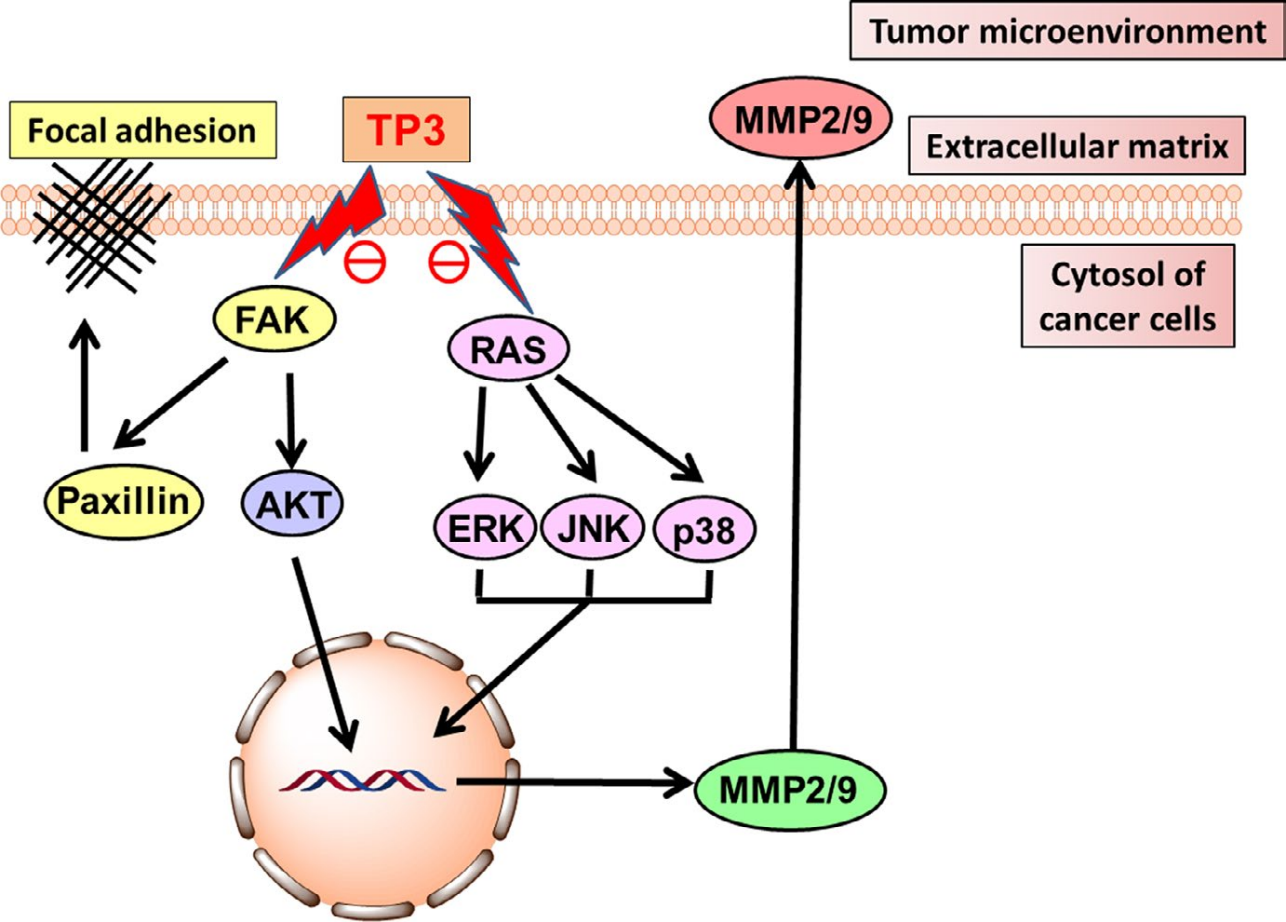
Proposed mechanisms through which TP3 inhibited GBM infiltration and mobility. Upon the administration of TP3, the ECM of the TME balance is disturbed through modulating the expression levels of MMPs, MAPK, FAK, and AKT pathways. p38, ERK, and JNK, the mitogen‐activated protein kinases; ECM, extracellular matrix; FAK, focal adhesion kinase; GBM, glioblastoma multiforme; MAPK, mitogen‐activated protein kinase; MMPs, matrix metalloproteinases; TME, tumor microenvironment; TP3, tilapia piscidin 3

Tilapia piscidin 3 has clearly shown to modulate the TME balance that led to the prevention of cell infiltration and mobility at different stages of the infiltration cascade. In intracranial infiltration, cancer cells are reorganized to change the cell‐to‐cell and cell‐to‐matrix adhesions.^8^ The loss of the adhesions allows malignant cells to morphologically polarize and to construct membrane protrusions, resulting in changes of size and dissociating from the primary tumor mass. This enables the cancer cells to reach forward, attach, and degrade the basement membrane of the ECM, which generates paths for further mobilization and migration. The ECM can also act as an anchor to help cancer cells migrate forward, through the rearrangement of mobility‐associated protein structures at FAs.^8^, ^54^ After the migrating cells reach likely points for settlement, they may reconstruct cell‐to‐cell and cell‐to‐matrix adhesions. Upon completion of the process, the intracranial invasion occurs with these infiltrated cancer cells that may proliferate and grow at the secondary foci. The molecular mechanisms of anti‐infiltration and anti‐mobility by TP3‐induced are shown in Figure 6. When TP3 acts on brain cancer cells, it inhibits RAS activity that subsequently ablates the phosphorylation of ERK, p38 and JNK. In addition, TP3 inhibits FAK activity, which results in the downregulation of AKT. The inhibition on both RAS and FAK leads to ablated secretion of MMP2 and MMP9 to the TME. The ECM degradation effects of MMP2 and MMP9 within the TME are therefore attenuated. Moreover, the TP3‐induced inhibition on FAK also affects paxillin, resulting in decreases in FAs.

Invasion of tumor cells is a complex process that is facilitated by a variety of factors, such as cell dynamics, the ECM, protease expression, junction proteins, altered expression of cytokines, and migration of cells to adjacent tissues.^54^ It has been reported that MMP2 and MMP9 cause the proteolysis of ECM components and induction of cancer cell invasion.^55^ Sidhu et al reported that exfoliated exosomes of cancer cells had an ECM metalloproteinase inducer that was delivered to fibroblasts and induced MMPs secretion. The MMPs alter the ECM via proteolysis to promote tumor cell invasion and metastasis.^56^ FAK has a central role in modulating the maturation and stability of FAs, while paxillin is a FA adaptor. Both proteins are involved in focal‐adhesion dynamics and cell migration. Mitogen‐activated protein kinases play a well‐known role in cell proliferation, carcinogenesis, differentiation, inflammation, and stress response, but there is increasing evidence that this family is also essential for cell migration and invasion function. In this study, we demonstrated for the first time that TP3 elicited migration and invasion dysfunction of glioblastoma cells through deceasing MMPs, MAPKs, and FAK pathways.

Benefited from the nature of peptides, TP3 is expected to be endowed with multiple advantages over small molecule and protein‐based drugs. These include the ease of synthesis and chemical modifications, low production cost and limited side‐effects due to no drug accumulation in tissues.^57^, ^58^ In addition, TP3 may show cytotoxicity selectivity for cancer cells over non‐cancer counterparts. This preference results from a fact that cancer cells display elevated levels of negatively‐charged phosphatidylserine on the cell membrane,^34^ which is in favor of positively charged TP3 to reach the malignantly transformed cells. To our best knowledge, our study first describes that the antimicrobial TP3 peptide has shown promise in the anti‐cancer application, and it can be considered as an anti‐cancer peptide. The promising anti‐cancer activities and advantageous peptide nature of TP3 together suggest that it merits further development as an anti‐brain cancer therapeutic.

## CONFLICT OF INTEREST

The authors declare no conflict of interest.

## AUTHOR CONTRIBUTIONS

N.‐F.C., H.‐M.K., Y.‐F.C., and P.‐C.S. contributed to conceptualization. Y.‐Y.L. contributed to data curation. P.‐C.S. contributed to methodology. S.‐J.T., H.‐T.L., and S.‐N.Y. contributed to formal analysis. H.‐M.K. contributed to investigation. H.‐T.L. and W.‐F.C. contributed to project administration. N.‐F.C. contributed to supervision. N.‐F.C. and H.‐M.K. contributed to validation. H.‐M.K. contributed to visualization. P.‐C.S. and Y.‐F.C. contributed to writing—original draft. N.‐F.C., H.‐M.K., and P.‐C.S contributed to writing—review and editing.

## Supporting information

Fig S1

Video S1

Supplementary Material

Supplementary Material

## Data Availability

The data that support the findings of this study are available from the corresponding author upon reasonable request.
