# Supplementary figures and images for: TP3, an antimicrobial peptide, inhibits infiltration and motility of glioblastoma cells via modulating the tumor microenvironment
Source: Cancer Med. 2020 Apr 7;9(11):3918–31. doi: 10.1002/cam4.3005 (PMC7286473; doi:10.1002/cam4.3005)

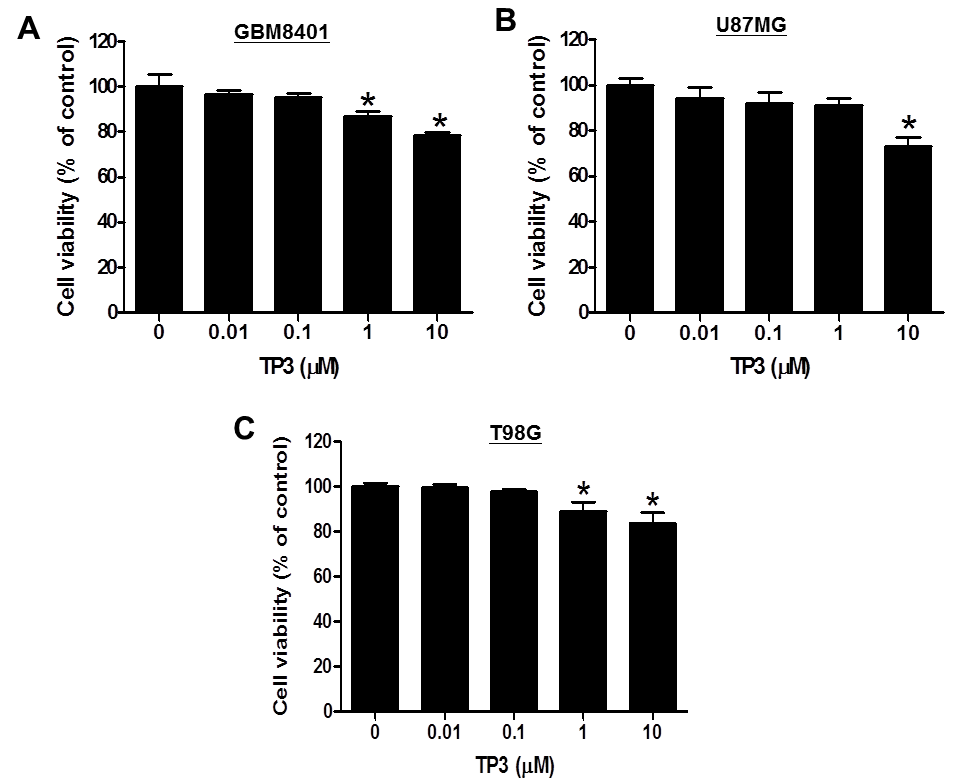

Supplement: Supplementary file 1 — Fig S1 [file CAM4-9-3918-s001.tiff]
