# Supplementary material for: TP3, an antimicrobial peptide, inhibits infiltration and motility of glioblastoma cells via modulating the tumor microenvironment
Source: Cancer Med. 2020 Apr 7;9(11):3918–31. doi: 10.1002/cam4.3005 (PMC7286473; doi:10.1002/cam4.3005)
Supplement: Supplementary file 3 — Supplementary Material [file CAM4-9-3918-s003.docx]

**TP3, an Antimicrobial Peptide, Inhibits Infiltration and Motility of Glioblastoma Cells via Modulating the Tumor Microenvironment**

**Ying-Fa Chen^1,2,†^, Po-Chang Shih^3,4,†^, Hsiao-Mei Kuo^4,5,†^, San-Nan Yang^6^, Yen-You Lin^7^, Wu-Fu Chen^4,8,9^, Shiow-Jyu Tzou^10,11^, Hsin-Tzu Liu^12^, Nan-Fu Chen^11,13,14^***

1. Department of Neurology, Kaohsiung Chang Gung Memorial Hospital and Chang Gung University College of Medicine, Kaohsiung 83301, Taiwan; alpha0716@gmail.com (Y.-F.C)

2. Center for Parkinson’s Disease, Kaohsiung Chang Gung Memorial Hospital and Chang Gung University College of Medicine, Kaohsiung 83301, Taiwan; alpha0716@gmail.com (Y.-F.C)

3. UCL School of Pharmacy, University College London, Bloomsbury, London WC1N 1AX, UK;

[po-chang.shih.14@ucl.ac.uk](mailto:po-chang.shih.14@ucl.ac.uk) (P.-C.S.)

4. Department of Marine Biotechnology and Resources, National Sun Yat-sen University, Kaohsiung, 80424, Taiwan; [hsiaomeikuo@gmail.com](mailto:hsiaomeikuo@gmail.com) (H.-M.K.); [po-chang.shih.14@ucl.ac.uk](mailto:po-chang.shih.14@ucl.ac.uk) (P.-C.S.); [ma4949@cgmh.org.tw](mailto:ma4949@cgmh.org.tw) (W.-F.C)

5. Center for Neuroscience, National Sun Yat-sen University, Kaohsiung, 80424, Taiwan; [hsiaomeikuo@gmail.com](mailto:hsiaomeikuo@gmail.com) (H.-M.K.)

6. Department of Internal Medicine, E-DA Hospital and College of Medicine, I-SHOU University, Kaohsiung, 84001, Taiwan; [y520729@gmail.com](mailto:y520729@gmail.com) (S.-N.Y.)

7. Department of Orthopedic Surgery, Ping-Tung Christian Hospital, Pingtung, 90059, Taiwan; [chas6119@gmail.com](mailto:chas6119@gmail.com) (Y.-Y.L.)

8. Department of Neurosurgery, Kaohsiung Chang Gung Memorial Hospital and Chang Gung University College of Medicine, Kaohsiung, 83301, Taiwan; [ma4949@cgmh.org.tw](mailto:ma4949@cgmh.org.tw) (W.-F.C)

9. Department of Neurosurgery, Xiamen Chang Gung Hospital, Xiamen, Fujian, China; [ma4949@cgmh.org.tw](mailto:ma4949@cgmh.org.tw) (W.-F.C)

10. Department of Nursing, Kaohsiung Armed Forces General Hospital, Kaohsiung, 80284, Taiwan; [jyu0120@gmail.com](mailto:jyu0120@gmail.com)

(S.-J.T.)

11. Institute of Medical Science and Technology, National Sun Yat-Sen University, Kaohsiung, Taiwan;

chen06688@gmail.com (N.-F.C.); jyu0120@gmail.com (S.-J.T.);

12. Department of Medical Research, Hualien Tzu Chi Hospital, Buddhist Tzu Chi Medical Foundation, Hualien,

Taiwan; [HTL1@ms43.hinet.net](mailto:HTL1@ms43.hinet.net) (H.-T. L.)

13. Division of Neurosurgery, Department of Surgery, Kaohsiung Armed Forces General Hospital, Kaohsiung, 80284,

Taiwan; [chen06688@gmail.com](mailto:chen06688@gmail.com) (N.-F.C.)

14. Department of Neurological Surgery, Tri-Service General Hospital, National Defense Medical Center, Taipei, 11490,

Taiwan; [chen06688@gmail.com](mailto:chen06688@gmail.com) (N.-F.C.)

**†**These authors contributed equally to this work.

*****Correspondence to: Nan-Fu Chen, Ph.D., Division of Neurosurgery, Department of Surgery, Kaohsiung Armed Forces General Hospital, Kaohsiung 80284, Taiwan.

E-mail: chen06688@gmail.com

TEL: +886-7-7494963

Fax: +886-7-7498281 Received: date; Accepted: date; Published: date

1. **SUPPLEMENTARY FIGURE**

**Supplementary Figure S1.** Effects of TP3 on cell viability of GBM8401, U87MG, and T98G cells. (**A**) GBM8401, (**B**) U87MG, and (C) T98G cells were treated with an increasing concentration of TP3 for 24 h, and then an MTT assay was performed to measure cell viability. Cell viability is the quantification of the number of live cells and is usually expressed as a percentage of the control. Each bar represents mean ± standard error deviation (SED). **p* < 0.05, relative to the control.

**2. SUPPLEMENTARY MATERIALS AND METHODS**

**2.1 Cell viability assay**

The proliferation potentials of TP3-treated glioblastoma cells were assessed in triplicate using the colorimetric MTT assay. The yellow MTT is reduced by mitochondrial succinate dehydrogenases of living cells to form purple formazan. The experimental procedures from our previous study were followed for this study (1). In brief, each condition was conducted in triplicate using 24-well plates (Nunc, Roskilde, Denmark) with the glioblastoma cells seeded at a density of 3 x 10^4^ and incubated overnight. Subsequently, a range of TP3 concentrations (0.01, 0.1, 1 and 10 μM) were employed to each well and incubated for 24 h. The treated cells were subjected to MTT stain (20 μL of 5 mg/mL per well) at the end of treatments. Following 2-4 h incubation for the MTT staining at 37 °C, supernatants were aspirated prior to the addition of dimethyl sulfoxide (DMSO) to dissolve formazan. The absorbance was recorded at 570 nm and determined using an ELISA plate reader (Dynatech Laboratories, Chantilly, VA, USA).

**References**

1. Kuo HM, Tseng CC, Chen NF, Tai MH, Hung HC, Feng CW, et al. MSP-4, an Antimicrobial

Peptide, Induces Apoptosis via Activation of Extrinsic Fas/FasL- and Intrinsic Mitochondria

Mediated Pathways in One Osteosarcoma Cell Line. Marine Drugs. 2018;16:8.
