# Supplementary material for: TP3, an antimicrobial peptide, inhibits infiltration and motility of glioblastoma cells via modulating the tumor microenvironment
Source: Cancer Med. 2020 Apr 7;9(11):3918–31. doi: 10.1002/cam4.3005 (PMC7286473; doi:10.1002/cam4.3005)
Supplement: Supplementary file 4 — Supplementary Material [file CAM4-9-3918-s004.docx]

**TP3, an Antimicrobial Peptide, Inhibits Infiltration and Motility of Glioblastoma Cells via Modulating the Tumor Microenvironment**

**Ying-Fa Chen^1,2,†^, Po-Chang Shih^3,4,†^, Hsiao-Mei Kuo^4,5,†^, San-Nan Yang^6^, Yen-You Lin^7^, Wu-Fu Chen^4,8,9^, Shiow-Jyu Tzou^10,11^, Hsin-Tzu Liu^12^, Nan-Fu Chen^11,13,14^***

1. Department of Neurology, Kaohsiung Chang Gung Memorial Hospital and Chang Gung University College of Medicine, Kaohsiung 83301, Taiwan; alpha0716@gmail.com (Y.-F.C)

2. Center for Parkinson’s Disease, Kaohsiung Chang Gung Memorial Hospital and Chang Gung University College of Medicine, Kaohsiung 83301, Taiwan; alpha0716@gmail.com (Y.-F.C)

3. UCL School of Pharmacy, University College London, Bloomsbury, London WC1N 1AX, UK;

[po-chang.shih.14@ucl.ac.uk](mailto:po-chang.shih.14@ucl.ac.uk) (P.-C.S.)

4. Department of Marine Biotechnology and Resources, National Sun Yat-sen University, Kaohsiung, 80424, Taiwan; [hsiaomeikuo@gmail.com](mailto:hsiaomeikuo@gmail.com) (H.-M.K.); [po-chang.shih.14@ucl.ac.uk](mailto:po-chang.shih.14@ucl.ac.uk) (P.-C.S.); [ma4949@cgmh.org.tw](mailto:ma4949@cgmh.org.tw) (W.-F.C)

5. Center for Neuroscience, National Sun Yat-sen University, Kaohsiung, 80424, Taiwan; [hsiaomeikuo@gmail.com](mailto:hsiaomeikuo@gmail.com) (H.-M.K.)

6. Department of Internal Medicine, E-DA Hospital and College of Medicine, I-SHOU University, Kaohsiung, 84001, Taiwan; [y520729@gmail.com](mailto:y520729@gmail.com) (S.-N.Y.)

7. Department of Orthopedic Surgery, Ping-Tung Christian Hospital, Pingtung, 90059, Taiwan; [chas6119@gmail.com](mailto:chas6119@gmail.com) (Y.-Y.L.)

8. Department of Neurosurgery, Kaohsiung Chang Gung Memorial Hospital and Chang Gung University College of Medicine, Kaohsiung, 83301, Taiwan; [ma4949@cgmh.org.tw](mailto:ma4949@cgmh.org.tw) (W.-F.C)

9. Department of Neurosurgery, Xiamen Chang Gung Hospital, Xiamen, Fujian, China; [ma4949@cgmh.org.tw](mailto:ma4949@cgmh.org.tw) (W.-F.C)

10. Department of Nursing, Kaohsiung Armed Forces General Hospital, Kaohsiung, 80284, Taiwan; [jyu0120@gmail.com](mailto:jyu0120@gmail.com)

(S.-J.T.)

11. Institute of Medical Science and Technology, National Sun Yat-Sen University, Kaohsiung, Taiwan;

chen06688@gmail.com (N.-F.C.); jyu0120@gmail.com (S.-J.T.);

12. Department of Medical Research, Hualien Tzu Chi Hospital, Buddhist Tzu Chi Medical Foundation, Hualien,

Taiwan; [HTL1@ms43.hinet.net](mailto:HTL1@ms43.hinet.net) (H.-T. L.)

13. Division of Neurosurgery, Department of Surgery, Kaohsiung Armed Forces General Hospital, Kaohsiung, 80284,

Taiwan; [chen06688@gmail.com](mailto:chen06688@gmail.com) (N.-F.C.)

14. Department of Neurological Surgery, Tri-Service General Hospital, National Defense Medical Center, Taipei, 11490,

Taiwan; [chen06688@gmail.com](mailto:chen06688@gmail.com) (N.-F.C.)

**†**These authors contributed equally to this work.

*****Correspondence to: Nan-Fu Chen, Ph.D., Division of Neurosurgery, Department of Surgery, Kaohsiung Armed Forces General Hospital, Kaohsiung 80284, Taiwan.

E-mail: chen06688@gmail.com

TEL: +886-7-7494963

Fax: +886-7-7498281 Received: date; Accepted: date; Published: date

**Supplementary Video S1.** Effects of TP3 on filopodia protrusions and cell attachment in GBM8401 cells, observed using live-cell tomographic microscopy. The GBM8401 cells were plated in a glass-bottomed 3.5 cm dish overnight, followed by filming immediately after 10 µM TP3 injections. GBM8401 cells were subjected to performing live cells time-lapse imaging experiments, capturing cell images once every 15 minutes for 5 h, and recording the dynamic changes of the edge extension. The GBM8401 cells stretched out their filopodia bodies actively under TP3-null condition. Upon TP3 administration, the filopodia bodies contracted inwards their cell bodies and began to shrink in size, resulting from the outermost cell surface collapsed which left the cell membrane to be ebb tide-like.
